# Supplementary material for: Identification and functional analysis of non-coding regulatory small RNA FenSr3 in Bacillus amyloliquefaciens LPB-18
Source: PeerJ. 2023 May 15;11:e15236. doi: 10.7717/peerj.15236 (PMC10194069; doi:10.7717/peerj.15236)
Supplement: Supplemental Information 4 [file peerj-11-15236-s004.zip › KO/CK-vs-T1_map/map01524.html]

KEGG PATHWAY: Platinum drug resistance - Reference pathway


|  |  |
| --- | --- |
| **Platinum drug resistance - Reference pathway** |  |

[
Pathway menu
| Organism menu
| Pathway entry
| Show description
| User data mapping
]

Platinum-based drugs cisplatin, carboplatin and oxaliplatin are widely used in the therapy of solid malignancies, including testicular, ovarian, head and neck, colorectal, bladder and lung cancers. The mechanism of action of Platinum-based drugs involves covalent binding to purine DNA bases, which primarily leads to cellular apoptosis. Their clinical success is, however, limited due to severe side effects and intrinsic or acquired resistance to the treatment. Platinum resistance could arise from decreased drug influx, increased drug efflux, intracellular detoxification by glutathione, etc., decreased binding (e.g., due to high intracellular pH), increased DNA repair, decreased mismatch repair, defective apoptosis, and altered oncogene expression.

|  |  |  |
| --- | --- | --- |
| Reference pathway | 184% 150% 122% 100% 82% 67% 55% | 图片下载 |
